# It’s about the patients: Practical antibiotic stewardship in outpatient settings in the United States

**DOI:** 10.3389/fmed.2022.901980

**Published:** 2022-07-27

**Authors:** Alpesh N. Amin, E. Patchen Dellinger, Glenn Harnett, Bryan D. Kraft, Kerry L. LaPlante, Frank LoVecchio, James A. McKinnell, Glenn Tillotson, Salisia Valentine

**Affiliations:** ^1^Department of Medicine, University of California, Irvine, Irvine, CA, United States; ^2^Department of Surgery, University of Washington, Seattle, WA, United States; ^3^No Resistance Consulting, Birmingham, AL, United States; ^4^Division of Pulmonary and Critical Care Medicine, Department of Medicine, Washington University School of Medicine, St. Louis, MO, United States; ^5^College of Pharmacy, University of Rhode Island, Kingston, RI, United States; ^6^Department of Emergency Medicine, Valleywise Health, Arizona State University, Phoenix, AZ, United States; ^7^Infectious Disease Clinical Outcomes Research Unit, Division of Infectious Disease, Lundquist Research Institute at Harbor-UCLA, Torrance, CA, United States; ^8^GST Micro LLC, North, VA, United States; ^9^American Family Care, Birmingham, AL, United States

**Keywords:** antibiotic stewardship, antimicrobial stewardship, therapeutic antibacterial agents, microbial drug resistance, pneumonia, infectious skin diseases, overprescribing, inappropriate prescribing

## Abstract

Antibiotic-resistant pathogens cause over 35,000 preventable deaths in the United States every year, and multiple strategies could decrease morbidity and mortality. As antibiotic stewardship requirements are being deployed for the outpatient setting, community providers are facing systematic challenges in implementing stewardship programs. Given that the vast majority of antibiotics are prescribed in the outpatient setting, there are endless opportunities to make a smart and informed choice when prescribing and to move the needle on antibiotic stewardship. Antibiotic stewardship in the community, or “smart prescribing” as we suggest, should factor in antibiotic efficacy, safety, local resistance rates, and overall cost, in addition to patient-specific factors and disease presentation, to arrive at an appropriate therapy. Here, we discuss some of the challenges, such as patient/parent pressure to prescribe, lack of data or resources for implementation, and a disconnect between guidelines and real-world practice, among others. We have assembled an easy-to-use best practice guide for providers in the outpatient setting who lack the time or resources to develop a plan or consult lengthy guidelines. We provide specific suggestions for antibiotic prescribing that align real-world clinical practice with best practices for antibiotic stewardship for two of the most common bacterial infections seen in the outpatient setting: community-acquired pneumonia and skin and soft-tissue infection. In addition, we discuss many ways that community providers, payors, and regulatory bodies can make antibiotic stewardship easier to implement and more streamlined in the outpatient setting.

## Introduction

Every year in the United States (US), antibiotic-resistant pathogens are implicated in at least 35,000 deaths and over 2.8 million infections (1). Fundamentals of antibiotic stewardship dictate that clinicians can reduce the impact of antibiotic resistance by carefully prescribing antibiotics only when needed, with the right drug, dosage, and duration (2). While hospital-based stewardship programs have demonstrated remarkable value and healthcare benefit, the expansion of stewardship to the outpatient setting—including primary care clinics, urgent care (UC) settings, and skilled nursing facilities—may be less successful unless consideration is given to the unique nature of outpatient healthcare. This article describes the scope of the problem with outpatient stewardship in the US and systematic challenges limiting implementation, offering some pragmatic solutions to facilitate implementation.

## What’s the problem?

**FIGURE 1:**
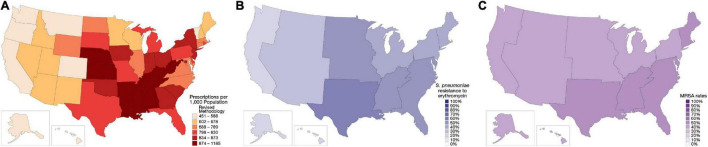
Regional distribution of antibiotic prescribing patterns and antibiotic resistance within the United States (US). **(A)** Outpatient antibiotic prescription rates from the Centers for Disease Control and Prevention, 2018 (3). **(B)** Erythromycin-resistant *Streptococcus pneumoniae* phenotype rates, 2019 (98). **(C)** Methicillin-resistant *Staphylococcus aureus* (MRSA) rates, as a percentage of all *S. aureus* isolates, 1997–2017 (51). Resistance rates were derived from isolates collected at US hospitals in the SENTRY surveillance program.

In the US in 2019, 250 million oral antibiotic prescriptions were written in the outpatient setting—roughly the equivalent of eight antibiotic prescriptions for every 10 people (Figure 1A) (3–5). One-third (∼47 million) of these outpatient antibiotic prescriptions are considered unnecessary (6). This is largely attributable to antibiotics prescribed for viral infections (e.g., viral upper respiratory infections, pharyngitis, and middle ear infections), as well as non-bacterial conditions such as allergy/asthma and bronchitis (7, 8).

Antibiotics for common acute infections are often prescribed for 10 or more days of therapy, which is longer than needed (9–11). At 129 Veteran’s Affairs medical centers, 40% of antibiotic prescriptions for pneumonia were for 8 days or longer (11). In a single-center study, 42% of uncomplicated skin infections treated in the ambulatory setting were prescribed antibiotic therapy for ≥ 10 days (9). Excessive antibiotic duration is associated with a higher risk of *Clostridioides difficile*–associated diarrhea and drug toxicity (12–14).

Depending on the infection type, some 25–50% of antibiotic prescriptions for bacterial infections do not align with current guidelines (6, 9, 15, 16) or may fail to adequately consider local resistance patterns. The current guidelines from the Infectious Disease Society of America and American Thoracic Society indicate that macrolide monotherapy is a first-line treatment option for the typical patient with community-acquired pneumonia (CAP; those with no comorbidities or risk factors for methicillin-resistant *Staphylococcus aureus* [MRSA] or *Pseudomonas aeruginosa*), but only if local *Streptococcus pneumoniae* resistance rates are < 25% (17). *S. pneumoniae* is resistant to macrolides in around 40–50% of isolates in the US, and most US regions exhibit resistance rates > 25% (Figure 1B) (18–21). Despite relatively clear guidance from the CAP guidelines and established patterns of antimicrobial resistance, azithromycin, a macrolide, remains the most commonly prescribed agent in the US, accounting for about 30–40% of outpatient CAP prescriptions written (22).

While some local public health agencies and health systems provide clinicians with local resistance information, these data are becoming more challenging to obtain (23). Furthermore, even if an antibiogram (i.e., antibiotic susceptibility test report) is available, primary care providers may benefit from expert interpretation of the data, including the data source and how they affect the risk/benefit decision for therapy. Antibiotic resistance profiles can differ substantially between isolates collected in the outpatient setting versus inpatient setting and, therefore, antibiograms produced by hospitals should be interpreted carefully when applied to outpatients.

## Why don’t we just have antibiotic stewardship in all outpatient settings?

Primary care physicians, advanced practice providers, and dentists account for the majority of outpatient antibiotic prescriptions written (24). Prescribers come from diverse specialties, geographic locations, and practice types (e.g., private vs. health system affiliates) (25). Implementation of effective antibiotic stewardship must be customized to each specific care setting and requires some expertise to establish. Moreover, for any substantial change in outpatient antibiotic use to be successfully implemented, outpatient clinicians need the resources and time to address inappropriate antibiotic prescribing.

Though antibiotic stewardship was originally introduced in inpatient care, regulatory bodies, and public health agencies are now implementing antimicrobial stewardship requirements in outpatient settings (26). The Centers for Disease Control and Prevention (CDC) adapted their inpatient stewardship recommendations to the outpatient setting, noting that clinicians should **demonstrate a commitment** to optimizing antibiotic prescribing and patient safety, take at least one **action for policy or practice** to improve antibiotic prescribing, **track prescribing practices** and **provide regular feedback** to clinicians, and provide **educational resources and expertise** on optimizing antibiotic prescribing (26). The Los Angeles County Department of Public Health has incorporated many of the CDC’s Core Elements of antibiotic stewardship into their Targeting Appropriate Prescribing in Outpatient Settings (TAP Out) program, which reduced inappropriate prescribing and provided well-received peer comparison reports on prescribing habits (27).

Recently, the Joint Commission, which is the largest healthcare accrediting body in the US, has been applying new antibiotic prescribing standards to accredited ambulatory healthcare (i.e., outpatient clinics, UC, or worksite clinics; Supplementary Table 1) (28, 29). One barrier to implementing antibiotic stewardship in outpatient settings is the lack of accountability for outpatient antibiotic stewardship through traditional regulatory bodies, i.e., the Centers for Medicare and Medicaid Services. Alternatively, payors may be able to play an important role in outpatient stewardship.

Several antibiotic stewardship programs have been developed specifically for implementation in skilled nursing facilities. For instance, the Agency for Health Care Quality created a four-part approach that includes methods to monitor and maintain a stewardship program (30). However, data from this program do not seem to have been published to date. Concurrently, one large health insurance organization has created its own antibiotic stewardship program, but again the effects are not publicly known (31). Full compliance at the participating sites may be difficult due to staffing shortages and lack of systems or protocols. While skilled nursing facilities have successfully implemented infection control measures (32), there is a need for more education and administrative oversight to fully implement the intended nature of antibiotic stewardship (33).

According to a 2018 Pew Trust report, almost 46% of antibiotic prescriptions written in the UC setting were unnecessary (24). These were mainly for respiratory tract infections. However, despite recent efforts by the Academy of Urgent Care Medicine, which developed an antibiotic stewardship education program, very few sites have completed the training to gain accreditation in antibiotic stewardship.

### “It’s not me”

Prescribers don’t think they’re part of the antibiotic prescribing problem. Almost all surveyed physicians say that, in general, there is a problem with antibiotic resistance and inappropriate prescribing in the US (34, 35). However, only about 50% of these surveyed physicians see the problem as occurring in their specific practice. This disconnect continues to fuel the problem, and we all need to accept responsibility and survey our prescribing habits.

### “I don’t have the data, and I don’t have the support to implement”

The average healthcare provider seeing patients in the community is not supported by health system-based education, interventions, and staff to guide appropriate prescribing practices. Therefore, the provider is left to navigate this complex field independently, sourcing guidelines and continuing education materials, and implementing stewardship practices. The prime example of this is the UC provider who usually works in isolation without regular peer-to-peer interaction, which is a crucial component of a successful antibiotic stewardship program.

### Guideline disconnect

National health agencies (the CDC) and professional organizations (Infectious Diseases Society of America) have published a variety of resources for clinicians on antibiotic prescribing, for particular infections and for more appropriate use of antibiotics in general (17, 36–42). However, the complexity of the documents, the length of time between document updates, and the inclusion of some content that doesn’t reflect real-world practice leads many community providers to turn instead to alternative resources, including decision support information sites such as UpToDate and Epocrates, or rely on their medical training (8, 43). Some of the guidelines lack specific recommendations on duration of therapy, therapy choice, or how to interpret local resistance patterns.

### Pressure to prescribe

Patients (and parents of young patients) often expect and may even pressure a provider for an antibiotic prescription when it is not indicated. About 84% of providers surveyed said they feel at least moderate pressure from patients for an antibiotic prescription (34). Patients’ and parents’ expectations for an antibiotic prescription can increase antibiotic prescribing (44). However, some of the perceived pressure from the perspective of the provider may not be the intention of the patient/parent, who instead is looking for reassurance and a better explanation of the management plan (2). For the independent practitioner in the outpatient setting, leaving the patient’s expectations unfulfilled risks having a “dissatisfied customer.” Some providers practice defensive prescribing of antibiotics, out of concern for missing bacterial infections and the possible medicolegal ramifications (45).

## Smart prescribing for outpatients

Here, we want to address smart prescribing for two of the most common bacterial infections seen in the outpatient setting, CAP and skin and soft-tissue infection (SSTI). For CAP and SSTI, several organizations have released updated clinical practice guidelines within the last 7 years (17, 38, 42). Despite the advances in therapeutic options, many prescribers in the outpatient setting are unaware of these updates or have not received continuing education about updates from previous guidelines.

### Community-acquired pneumonia

*S. pneumoniae* is the most commonly isolated bacterial pathogen in patients with pneumonia without underlying chronic lung disease; other causative pathogens include *Haemophilus influenzae*, *Mycoplasma pneumoniae*, *S. aureus*, and *Legionella pneumophila* (21, 46). A bacterial pathogen is isolated in about 25–50% of CAP cases, with many patients having no pathogen detected, and viral pathogens occurring in some cases (46, 47).

**FIGURE 2:**
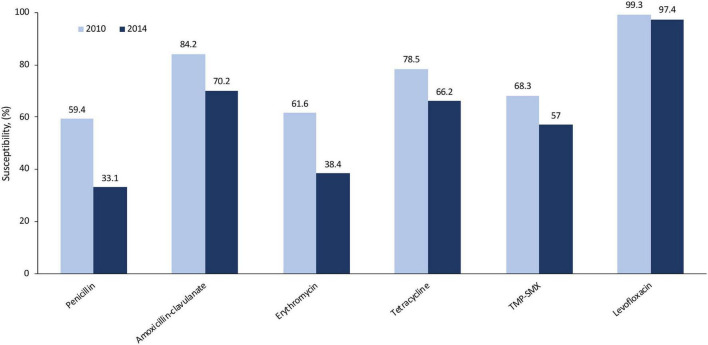
Susceptibility rates of *Streptococcus pneumoniae* to common antibiotics in North America (2010, 2014) using CLSI breakpoints (20). Amoxicillin–clavulanate rates were determined using non-meningitis breakpoints. CLSI, Clinical and Laboratory Standards Institute; TMP/SMX, trimethoprim–sulfamethoxazole.

In the community and UC/emergency department settings, the most commonly prescribed antibiotics for CAP are azithromycin and fluoroquinolones, accounting for 50–66% of all prescriptions for CAP treatment (7, 11, 22). While many providers prescribe an antibiotic empirically for CAP, local data on pathogens and susceptibility (if available) could better inform the treatment approach. From a robust collection of isolates from North America, the susceptibility rates of *S. pneumoniae* to levofloxacin were high (97–99%) and remained stable from 2010 to 2014. There was a decrease in susceptibility rates over this period for other common antibiotics, such as amoxicillin, erythromycin, tetracycline (which can be used as a surrogate for doxycycline susceptibility), and trimethoprim–sulfamethoxazole (also known as co-trimoxazole; Figure 2), which may have the potential to render these agents less appropriate for empiric treatment of CAP (20). More recent studies show that resistance rates of *S. pneumoniae* to macrolides (e.g., azithromycin) are approximately 40–50% in the US (18, 19, 21). Inappropriate use of antibiotics can lead to selection of resistant mutants either within a class or, less commonly, with other agents, known as co-resistance. Thus, such collateral damage has to be considered. Based on national rates of antimicrobial resistance to *S. pneumoniae*, azithromycin monotherapy for CAP is not recommended. Lack of specificity in our national guidelines leaves most providers guessing at best available therapy rather than following expert guidance.

### Skin infection

*S. aureus* is the most commonly isolated pathogen from SSTIs, with Group A streptococci and *P. aeruginosa* also found to a lesser extent (48, 49). About half of all *S. aureus* isolates from SSTI cases in the US are MRSA strains (Figure 1C) (49, 50). Gram-negative pathogens, when they occur in SSTI, are more likely to be associated with surgical-site infections of the abdominal wall, or infections in the anal and perineal region (49).

**FIGURE 3:**
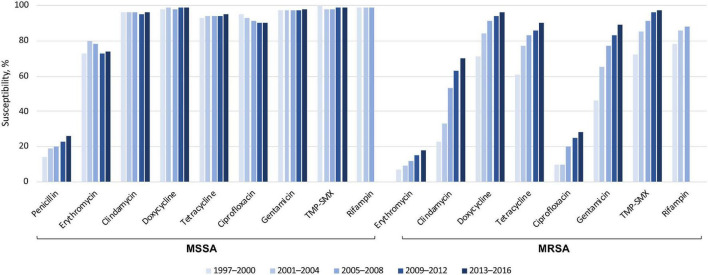
Susceptibility of > 191,000 *S. aureus* isolates to older antibiotics, from a global surveillance program (51). MRSA, methicillin-resistant *Staphylococcus aureus*; MSSA, methicillin-susceptible *S. aureus*; TMP-SMX, trimethoprim–sulfamethoxazole.

Global susceptibility of *S. aureus* isolates between 1997 and 2016 showed susceptibility of methicillin-susceptible *S. aureus* (MSSA) isolates to many older agents was > 95%, except for penicillin and erythromycin (Figure 3) (51). The susceptibility of MRSA to these older antibiotics was generally lower than methicillin-susceptible *S. aureus*. However, the susceptibility rates did increase over the last two decades, possibly as a result of the spread of MRSA clones that are more susceptible to these agents. Many of the more recently approved antibiotics demonstrated susceptibility rates of > 99% against MRSA, except for levofloxacin (23% susceptible, from 72,000 isolates), delafloxacin (74% susceptible, from > 10,000 isolates), and ceftaroline (92% susceptible, from > 40,000 isolates).

Uncomplicated (superficial), purulent SSTIs can often be treated by incision and drainage alone, while non-purulent SSTIs require antibiotics (38, 39, 52, 53). Antibiotic therapy when added to incision and drainage for abscesses can lead to a moderate improvement in efficacy (in one randomized study: 82–83% clinical success, depending on which antibiotic regimen was selected, vs. 68.9% in the incision and drainage only group), though this improvement may be limited to patients who have a positive culture for *S. aureus* (53). Antibiotic therapy for SSTI often consists of cephalexin and/or trimethoprim–sulfamethoxazole, though different agents may be used by provider choice and for certain types of infections (9, 54, 55). If MRSA is known or suspected to be present in the lesion, guideline-recommended treatments include vancomycin, linezolid, clindamycin, daptomycin, ceftaroline, doxycycline, minocycline, and trimethoprim–sulfamethoxazole; of note, cephalexin is not a recommended agent for treating known or suspected MRSA infections (38, 40, 42). SSTIs are often treated in the UC/emergency department setting, where providers may justifiably err on the side of treating with antibiotics because of the episodic nature of patient care and the lack of follow-up. This episodic nature of care occurs for many patients in the US with various conditions, including other infectious diseases, and may not readily be addressable without a systemic change in the availability and interconnectedness of electronic medical records (EMRs) or in the architecture of healthcare delivery and reimbursement.

## Smart prescribing in the outpatient setting

Given the number of infections that occur annually for CAP and SSTI in the US, there are millions of chances to make a smart and informed choice when prescribing antibiotics. Antibiotic stewardship in the community, or “smart prescribing” as we suggest, should factor in antibiotic efficacy, safety, local resistance rates, and overall cost, in addition to patient-specific factors and disease presentation, to arrive at an appropriate therapy.

Almost half of surveyed providers said they would need “a lot of help” to implement antibiotic stewardship practices (34). We recognize the magnitude of the challenge and have assembled this easy-to-use best practice guide for providers in the outpatient setting who lack the time or resources to develop a plan or consult lengthy guidelines.

### Measure existing prescribing habits

From EMR prescribing data, providers can identify one or two issues within their practice to address (e.g., inappropriate prescribing for a particular diagnosis code; peer benchmarking for antibiotic duration and dosing), and determine what action to take (56–58). Providers can then monitor the issue(s) periodically (e.g., monthly) to see if the data are improving (59). To obtain an approximate idea of how many patients fail initial treatment, providers can examine antibiotic refills, antibiotic switches, emergency department visits, and hospitalizations within 30 days of the initial prescription, though these data may be limited by the interconnectedness of EMRs or the patient obtaining all of their care within one health system. In the absence of an EMR, providers could review a patient’s recent medical history to determine previous treatments, and treatment failures on an individual basis. Other data and aggregate analyses require electronic systems and knowledge to interpret the results.

### Choose an appropriate drug, dose, and duration

Recommendations are provided for the most common pathogens and patient populations in CAP (Table 1) and SSTI (Table 2). These recommendations are for the “standard” patient with one of these bacterial infections; a good rule of thumb is that for ∼80% of cases, your treatment should fall along these lines.

**TABLE 1:** Smart prescribing recommendations for community-acquired pneumonia.

General recommendations	
Duration of treatment	• Initial duration of antibiotic treatment should be 5–7 days (10)
	• Short course associated with fewer adverse reactions (12)
	• Evidence in CAP (84–88)
Choice of treatment	• Choose antibiotic based on local resistance patterns, known/suspected pathogen; national resistance rates are suitable alternative
	• If local macrolide resistance rates are unknown, choose other first-line monotherapy (89)
	• If local rates are known to be < 25%, can consider a macrolide
	• Informed by prior microbiological culture if available; revised when microbiological culture is available
	• Common treatments to consider: beta-lactams + macrolides, tetracyclines, fluoroquinolones

**Reasons to deviate**	**What to change**

Recent antibiotic use	Do not repeat recent drug; increased likelihood that the pathogen is resistant to the specific antibiotic
Drug Resistance in Pneumonia (DRiP) score ≥ 4*	Likely need for extended-spectrum antibiotics (90)
Structural lung disease (e.g., fibrosis, lung cancer)	Risk factor for *Pseudomonas aeruginosa*
Lung cancer, post-obstructive pneumonia	Consider longer therapy duration
Exposure to birds, farm animals, water reservoirs	Risk factors for atypical pathogens
Immunocompromised	Consider longer therapy duration

*DRiP score calculation: antibiotic use within 60 days (2 points); residence in long-term care facility (2 points); tube feeding (2 points); infection with drug-resistant pathogen within 1 year (2 points); hospitalization within 60 days (1 point); chronic pulmonary disease (1 point); poor functional status (1 point); gastric acid suppression (1 point); wound care (1 point); methicillin-resistant Staphylococcus aureus colonization within 1 year (1 point) (90). CAP, community-acquired pneumonia.

**TABLE 2:** Smart prescribing recommendations for skin infection.

General recommendations	
Duration of treatment	• Initial duration of antibiotic treatment should be 5–7 days (10)
	• Short course associated with fewer adverse reactions (12)
	• Evidence in SSTI (91–94)
Choice of treatment	• Incision and drainage is encouraged when clinically indicated, followed by culture
	• May be sufficient to resolve superficial infection (38, 39, 52, 53)
	• Choose antibiotic based on local resistance patterns, known/suspected pathogen; national resistance rates are suitable alternative
	• Common treatments to consider: cephalosporins (not for MRSA), sulfonamides, glycopeptides, oxazolidinones, tetracyclines

**Reasons to deviate**	**What to change**

Recent antibiotic use	Do not repeat recent drug; increased likelihood that the pathogen is resistant to the specific antibiotic
Lymphedema	Coverage for Group A streptococci; longer therapy duration (95)
Picking at skin	Educate patient about handwashing and avoiding lesion(s)
Injection drug use	*Staphylococcus*, streptococci (including oral origin), and anaerobes more likely (40, 96, 97)
Lesion below the waist	Coverage for Gram-negative rods more likely needed (42)
Lesion on hand or face	Surgical referral urgently, treat more aggressively than other anatomical locations
Immunocompromised	Consider longer therapy duration

MRSA, methicillin-resistant Staphylococcus aureus; SSTI, skin and soft-tissue infection.

Community prescribing tends to follow standard dosing of antibiotics, but providers should be aware of the potential need for dose adjustments, for example related to body size or comorbid conditions (e.g., renal or hepatic impairment). For some patients, providers will need to take a different approach to antibiotic treatment based on certain patient or infection factors (Tables 1, 2). In all cases, providers should use their best judgment, tailor their treatment choice to each patient (their medical history, presentation, comorbid conditions, risk factors, and lifestyle), and use a shorter course whenever possible. Additionally, providers should watch out for certain safety issues that would suggest choosing an alternate antibiotic (Supplementary Table 2).

Delayed prescribing (“watchful waiting”) may assist in avoiding inappropriate prescribing related to patient pressure to prescribe, and thus reduce antibiotic resistance, by advising patients to return if symptoms do not improve within a few days or worsen (2, 60). It can also be a useful tool to allay a patient’s concerns that they present with at the initial visit. For UC settings, providers can offer the patient an antibiotic prescription with specific directions to fill it only if their symptoms haven’t improved in a few days, or write a future date on the prescription to be filled under the same circumstances. In cases where clinicians are uncertain of infections, a delayed prescription may be an appropriate safety net.

### Case management

Ideally, a nurse or case manager should follow up with the patient at Day 2–3 after beginning antibiotic treatment to see if there are signs of an early response to treatment or any worsening symptoms. However, additional staffing may be needed to achieve this, which might be difficult to implement in certain practices. Alternatively, groups of providers can hold regular debriefing sessions to discuss cases and note any patterns of disease presentation or treatment failure. For patients with a skin infection, the provider can draw a circle around the initial extent of the infection and instruct the patient to call or send a photo of the lesion size at Day 1–2. This protocol also aids a second provider who sees the patient to assess the treatment response.

## Making smart prescribing easier

### Simplify guidance documents

Providers need guidance from experts that is easy to find and use, and reflects the real-world scenarios that they are faced with every day (43).

### Know your local resistance patterns

Ask your local health department or community hospital for information (61). If those resources can’t routinely provide this information, reach out for help to a local infectious disease specialist, who can be found at the hospital or through a local chapter of the Infectious Diseases Society of America, or to the laboratory where you send your routine culture data. Laboratories that are accredited by the College of American Pathologists are required to publish an annual antibiogram.

### Rapid assessments

Rapid diagnostic tests are available for various viral and bacterial pathogens for respiratory, gastrointestinal, sexually transmitted, and central nervous system infections, most of which provide results within 15–45 min (62). Using highly sensitive molecular diagnostic tests can significantly reduce unnecessary testing and treatment, including inappropriate antibiotic prescribing, though the results vary by pathogen and disease state. However, rapid diagnostic tests are often reimbursed in a flat fee payment per patient for outpatient providers. As such, the significantly higher costs of molecular and polymerase chain reaction testing must be absorbed by the provider. Unfortunately, this is not an economically feasible option. Diagnostic stewardship is likely a route to ensuring tests are undertaken in the appropriate patient and that the rapid accurate results assist with case management. Payers should be made aware of this situation and that the use of “expensive” tests upfront can reduce costs in the longer term.

Additional challenges to practical implementation of rapid assessments are sensitivity of the test and time and staff required to train and perform quality control of the test. Providers should also be aware that bacterial colonization (rather than infection) can return a positive result based on highly sensitive molecular diagnostic tests, which would not routinely warrant antibiotic treatment.

### Patient/parent education

Suppose providers feel that the patient or parent is expecting an antibiotic prescription. In that case, the provider can explain why an antibiotic isn’t needed and give other actionable treatment advice so the patient/parent feels that they walked away from the visit with useful information (Supplementary Table 3) (2, 63). Even simple interventions, such as clinicians posting an informational letter in examination rooms with a signed commitment to use antibiotics appropriately, can reduce inappropriate prescribing by 20% (Supplementary Figure 1 shows an example) (64). The CDC has many handouts, posters, and web images, in English and Spanish, from the “Be Antibiotics Aware” campaign that can be shared with patients and caregivers (65). One effective example is the “Viruses or Bacteria: What’s got you sick?” poster, which shows a checklist of common conditions and whether an antibiotic is indicated or not (Supplementary Figure 2). When appropriate, hand your patient one of the CDC’s “prescription” sheets for symptom relief of common cold/viral illness (66). Providers can also obtain training that’s been specifically designed around improving their communication skills regarding antibiotic prescribing (67).

If a patient needs an antibiotic, encourage them to adhere to dosing instructions, and explain why this is important. In some situations, it may be worth explaining why you are prescribing a specific antibiotic (e.g., a narrower-spectrum vs. a broader-spectrum one) for the patient’s infection. In all cases, let the patient know about the likely disease course with treatment, potential side effects, and when to follow up.

### Market the practice as accredited for antibiotic stewardship

The College of Urgent Care Medicine offers an Antibiotic Stewardship Commendation to practices that provide evidence of their compliance with the CDC’s Core Elements (68). Practices that receive this accreditation can advertise their achievement in their clinic and online.

### Automated systems

EMR systems can be useful tools toward better antibiotic prescribing practices. In addition to making data collection easier (what was prescribed for a particular diagnosis), the EMR system can include prompts for particular interventions, automatically populated fields that comply with current guidelines, and step-through decision making (69). Unfortunately, the financial and logistical hurdles to implement these features in an EMR may be too high for smaller practices to overcome.

### Provider behavioral change

In addition to the concern for missing an infection and the possible consequences (e.g., patient morbidity/mortality and litigation), diagnostic uncertainty drives a substantial amount of unnecessary antibiotic prescribing (70). There is an inherent contradiction between avoiding the downstream consequences of failed therapy and limiting inappropriate prescribing of antibiotics. While some internal factors that motivate providers’ prescribing habits would be difficult to change without a larger overhaul of the US healthcare system and law reform, some efforts can affect behavioral change in inappropriate antibiotic prescribing. Programs aimed at re-educating healthcare providers on appropriate antibiotic prescribing, providing individualized feedback, and peer comparisons can significantly reduce inappropriate prescribing (57, 71). For example, a recent study in a rural community setting included physician education through presentations on antibiotic stewardship and appropriate, guideline-concordant prescribing; feedback emails on guideline-discordant prescribing for a particular indication; and recommendations on how physicians could improve their prescribing. Additionally, patient education materials were distributed to clinics, from the CDC’s “Be Antibiotics Aware” campaign (71). This resulted in an absolute decrease of ∼15% in inappropriate prescribing during the 6-month influenza season. A randomized controlled trial of three different types of antibiotic prescribing interventions in primary care (*N* = 248 clinicians) found that the most significant reductions in inappropriate prescribing occurred after the providers (1) had to include written justification in the patient’s EMR for why the prescription was necessary, becoming a permanent part of the record; and (2) received regularly updated rankings of their prescribing rate compared with that of the top-performing peers (58, 72).

### Risk stratification in community-acquired pneumonia

Common laboratory tests, such as complete blood counts and basic metabolic profiles, can be used to generate a risk score for adults that is highly predictive of 30-day all-cause death (73). Disease-specific risk scores, such as the Pneumonia Severity Index or the Confusion, Urea nitrogen, Respiratory rate, and Blood pressure (CURB) score (or alternatively a CRB65 score), can identify adult patients considered low risk who may be suitable candidates for outpatient therapy, and patients at high risk of death who require inpatient treatment and follow-up (74).

## Controversies

Costs are part of the bigger picture of antibiotic treatment. In the outpatient setting, typically the only cost limit to the antibiotic is whether the patient’s health insurance will cover the prescription and if the patient can afford the co-pay. In the bigger picture of healthcare and societal costs of infections, while a patient may initially have an inexpensive treatment with an oral generic antibiotic for their particular infection, if the patient experiences treatment failure (potentially due to inappropriate drug, dose, or duration), then the overall cost of treating that infection escalates significantly (75). Cost savings unquestionably come into play when deciding between intravenous and oral drugs, thereby decreasing or eliminating inpatient or outpatient parenteral antibiotic therapy costs (76, 77).

While there have been several new antibiotics developed in the last decade, their use is often limited by institutional policies that they should be “saved” for special/last-resort use (78, 79). In practice, this can have the unintended consequence that non-ideal antibiotics are prescribed instead, potentially adding fuel to the fire of antibiotic resistance. Though there is a push by regulatory bodies to develop new antibiotics to combat antibiotic resistance threats, antibiotic stewardship practices may actually be having a negative effect on the research and development pipeline (1, 78, 80). So, we are left to wonder, what is an appropriate place in infection management for newer agents that have less acquired resistance or were designed to overcome common resistance mechanisms (79, 81)?

## Conclusion

Regardless of the treatment setting where it is implemented, antibiotic stewardship is an evolving field (82, 83). Community prescribers can help move the needle on antibiotic stewardship by keeping in mind the “4 Ds”: prescribe an antibiotic for a bacterial infectious Disease, with the appropriate Drug, Dose, and Duration. To truly make headway with smart prescribing in the outpatient setting, more help from public health agencies, regulatory bodies, and payors is needed to provide education, practical support for implementation, and financial incentives for smart prescribing, as well as guidance from a multidisciplinary group on a pragmatic approach to appropriate antibiotic use in the community.

## Author contributions

All authors contributed to data interpretation and reviewing/editing the manuscript and approved the final version to be published and were accountable for the work.

## Abbreviations

CAP: community-acquired pneumonia
CDC: Centers for Disease Control and Prevention
EMR: electronic medical record
MRSA: methicillin-resistant *Staphylococcus aureus*
SSTI: skin and soft-tissue infection
UC: urgent care
US: United States.
