# Supplementary material for: It’s about the patients: Practical antibiotic stewardship in outpatient settings in the United States
Source: Front Med (Lausanne). 2022 Jul 27;9:901980. doi: 10.3389/fmed.2022.901980 (PMC9363693; doi:10.3389/fmed.2022.901980)
Supplement: Supplementary file 1 [file Data_Sheet_1.docx]

Supplementary Material

# SUPPLEMENTARY TABLES

**Supplementary Table 1.** Summary of the Joint Commission Antimicrobial Stewardship Requirements (1).

| Requirement | Rationale |
| --- | --- |
| Identify an individual responsible for developing, implementing, and monitoring activities to promote appropriate antibiotic prescribing | - Antimicrobial resistance is growing, improving use of antibiotics is a patient safety priority - Identifying an individual increases likelihood of success - Antimicrobial stewardship may be primary job or in addition to other duties |
| Organization sets ≥1 antibiotic stewardship goal each year | - Allows the organization to focus efforts - Can review own prescribing data or consult national data to identify areas for focus - Organization’s protocols and guidelines, education resources, and measurement activities should align with goal |
| Organization uses evidence-based practice guidelines related to antibiotic stewardship goal(s) | - Using protocols and guidelines will help ensure that antibiotics are given to only patients who need them |
| Organization provides all clinical staff with educational resources related to the goal, and strategies that promote appropriate antibiotic prescribing | - Clinical staff need education about the goal so that patients receive clear and consistent recommendations when antibiotics are not indicated - Education can include recommended prescribing practices, and strategies to explain why some patients may not need an antibiotic |
| Organization collects, analyzes, and reports data on the goal to organizational leadership and prescribers | - Important to evaluate whether activities are successful, and share any progress with leadership - Can involve electronic or manual data collection, analysis methods - Sustained effort is often required to see change in culture of antibiotic prescribing |

**Supplementary Table 2.** Safety-related factors that warrant careful consideration of antibiotic choice in outpatient treatment of pneumonia or skin infection (2).

| **Antibiotic** | **Risk factor** |
| --- | --- |
| Fluoroquinolones | - QTc prolongation - Tendinopathy/tendon rupture - Neuropathy - Risk for *C. difficile infection* (esp. age ≥65 years, recent antibiotic use) |
| Cephalosporins | - Risk for *C. difficile infection* (esp. age ≥65 years, recent antibiotic use) |
| Penicillins | - Hypersensitivity reaction |
| Clindamycin | - Risk for *C. difficile infection* (esp. age ≥65 years, recent antibiotic use) - Hematologic or renal issues with prolonged therapy |
| Tetracyclines | - Developmental effects (avoid in children, pregnancy) - Photosensitivity |
| Oxazolidinones | - Serotonin syndrome (esp. with prior or concomitant serotonergic agents) - Myelosuppression, visual issues with prolonged therapy |
| Macrolides | - QTc prolongation |
| Aminoglycosides | - Nephrotoxicity - Ototoxicity |

**Supplementary Table 3.** Managing patient/parent expectations for antibiotic prescription.

| **When…** | **What to say…** |
| --- | --- |
| Non-antibiotic treatments are indicated | “Drink extra fluids.”  “A humidifier or salt water nose spray can help congestion.”  “Popsicles or throat lozenges can help a sore throat.” [Lozenges for patients ≥4 years]  “Honey can help a cough.” [Honey for patients ≥1 year]  “Sleep with an extra pillow or towel to prop up your head, to relieve head pressure or coughing at night.” |
| Making plans for follow-up | “If you’re not feeling better in 3–4 days, call my office and we’ll see if we need to change the plan.”  “If your child is not better in 1 week or starts a fever, come back in.” |
| Antibiotics are not indicated | “Your sickness is caused by a virus, and antibiotics don’t work on viruses.”  “Antibiotics won’t help you feel better any sooner, and sometimes they can have side effects that we want to avoid if possible.” |
| Delayed prescribing | “Your child has an ear infection that should clear up on its own. In case it doesn’t, you can fill this antibiotic prescription in 2 days if your child is not feeling better or is getting worse.” |

Adapted from Fleming-Dutra, et al (3).

# SUPPLEMENTARY FIGURES

**
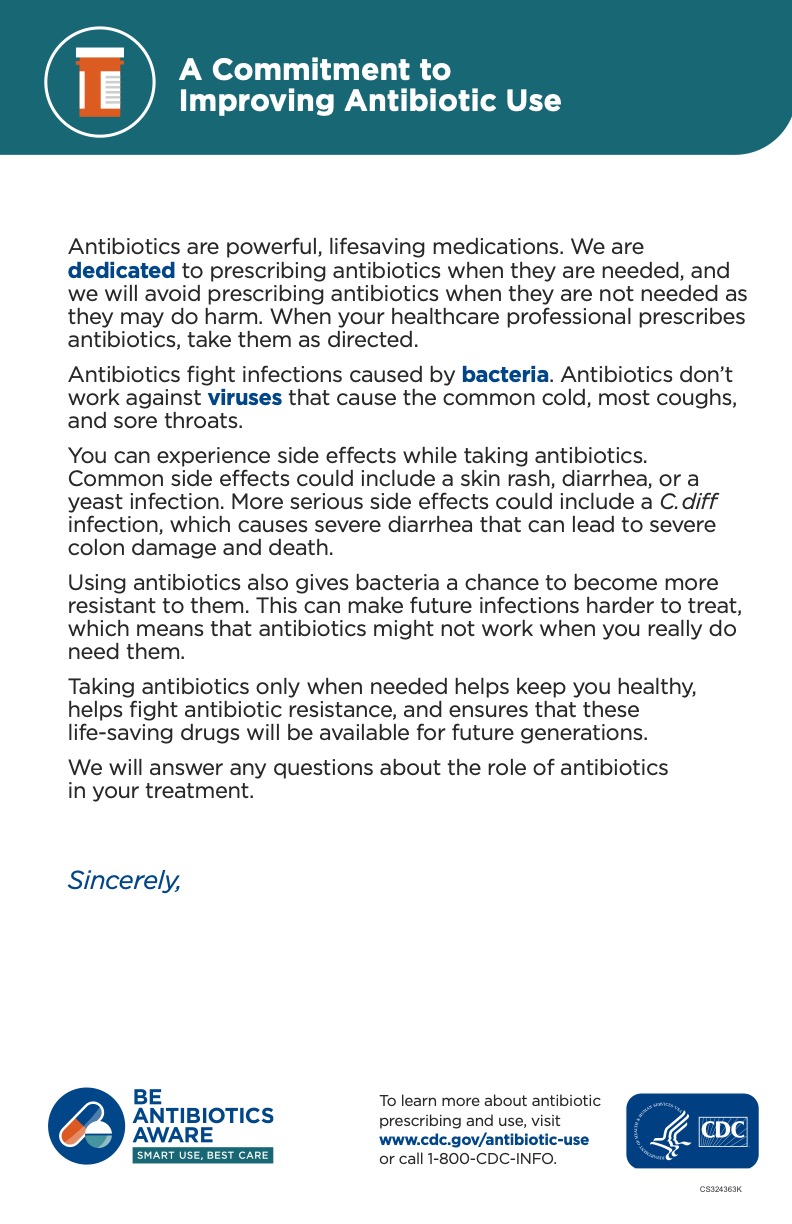
**

**Supplementary Figure 1.** Example of signed commitment letter to post in the clinic for appropriate antibiotic use. From the Centers for Disease Control and Prevention (4).

| **Viruses or Bacteria**  What’s got you sick? | | | | |
| --- | --- | --- | --- | --- |
| **Illness** | **Usually Caused By…** | | | **Will Antibiotics Help?** |
|  | Bacteria | Bacteria or Virus | Virus |  |
| Strep throat | ✓ |  |  | **Yes** |
| Urinary tract infection | ✓ |  |  | **Yes** |
| Whooping cough | ✓ |  |  | **Yes** |
| Sinus infection |  | ✓ |  | **Usually not**  Antibiotic need depends on symptoms, duration |
| Middle ear infection |  | ✓ |  | **Usually not**  Most clear on their own  Swimmer’s ear can clear with ear drops |
| Chest cold/ bronchitis* |  | ✓ |  | **Usually not**  Most clear on their own |
| Head cold/runny nose |  |  | ✓ | **No** |
| Sore throat (not Strep) |  |  | ✓ | **No** |
| Flu |  |  | ✓ | **No** |

**Supplementary Figure 2.** Printable poster for the clinic.

*In otherwise healthy children and adults.

Modified from the CDC’s Be Antibiotics Aware Campaign Materials to provide more specific, yet still concise, information for the patient (5).

# REFERENCES

1. The Joint Commission. R3 Report, Issue 23: Antimicrobial Stewardship in Ambulatory Health Care (2019). <https://www.jointcommission.org/standards/r3-report/r3-report-issue-23-antimicrobial-stewardship-in-ambulatory-health-care/>. [Accessed December 14, 2021]
2. Lee GC, Burgess DS. “Antimicrobial regimen selection,”. In: DiPiro JT, Talbert RL, Yee GC, et al., editors. Pharmacotherapy: A Pathophysiologic Approach. New York, NY: McGraw Hill Education (2017). P. 1731-1742.
3. Fleming-Dutra KE, Mangione-Smith R, Hicks LA. How to prescribe fewer unnecessary antibiotics: Talking points that work. *Am Fam Physician*. (2016) 94:200-2.
4. Centers for Disease Control and Prevention. <https://www.cdc.gov/antibiotic-use/pdfs/Commitment-Poster-english-11x17-P.pdf> [Accessed December 14, 2021].
5. Centers for Disease Control and Prevention Be Antibiotics Aware Campaign Materials. <https://www.cdc.gov/antibiotic-use/materials-references/index.html> [Accessed December 14, 2021].
